# Obstetrical Clinique

**Published:** 1851-01

**Authors:** 


*Obstetrical Clinique.*—An Obstetrical Clinique has recently been instituted by Prof. Bedford, at the University of the city of New York. This is a novelty, if not an innovation in medical instruction, for which we think Prof. B. is entitled to credit.

By the way, it is gratifying to see that the senseless clamor of some, on the subject of College Cliniques has died away. They are not substitutes for proper clinical, i. e., hospital instruction, but they possess a certain share of the value which belongs to the latter. Students cannot see too much. Illustration gives not only interest, but practical efficiency to medical teaching. A person may be said to have *learned* what has been rendered intelligible to his understanding, but he cannot be said to *know* the truths of natural science, until his senses have taken cognizance of the phenomena to which these truths relate. Demonstrative instruction in all the departments of medicine, is, therefore, desirable. Obstetrics has suffered more than other branches, hitherto, in this country, for the want of this advantage, but we augur favorably for an improvement from the plan adopted by Prof. Bedford.

